# Coexistence of Sympatric Large Carnivores: Spatio‐Temporal Interactions Between Tigers and Leopards in Parsa National Park, Nepal

**DOI:** 10.1002/ece3.71547

**Published:** 2025-06-21

**Authors:** Dol Raj Thanet, Pramod Raj Regmi, Babu Ram Lamichhane, Dipanjan Naha, Caitlin Kupferman, James C. Beasley, Mandip Pangeni, Anil Shrestha, Saneer Lamichhane, Haribhadra Acharya, Bed Kumar Dhakal, Bhagawan Raj Dahal, Chiranjibi Prasad Pokheral, Naresh Subedi

**Affiliations:** ^1^ Institute of Forestry, Hetauda Campus Tribhuvan University Hetauda Nepal; ^2^ National Trust for Nature Conservation Lalitpur Nepal; ^3^ Wildlife Conservation and Research Endeavor (WILD CARE) Nepal Lalitpur Nepal; ^4^ Savannah River Ecology Laboratory University of Georgia Aiken South Carolina USA; ^5^ Department of National Parks and Wildlife Conservation Kathmandu Nepal; ^6^ Faculty of Forestry University of British Columbia Vancouver Canada; ^7^ Department of Wildlife Ecology and Conservation, School of Natural Resources and Environment University of Florida Gainesville Florida USA; ^8^ Zoological Society of London Nepal Office Kathmandu Nepal

**Keywords:** activity patterns, camera trap, co‐occurrence, occupancy model, *Panthera pardus*, *Panthera tigris*, spatial capture‐recapture model

## Abstract

Understanding interspecific interactions between tigers ( *Panthera tigris* ) and leopards ( *Panthera pardus* ) is crucial for effective conservation planning. However, most studies have been conducted only in well‐known protected areas, leaving knowledge gaps in other parts of their overlapping range. This study investigates the spatial‐temporal interactions between sympatric carnivores (tigers and leopards) in Parsa National Park (PNP), Nepal. Camera trap data obtained from 157 sampling sites (2 × 2 km grid cells) were used to assess daily temporal activity patterns, single‐species occupancy, and density of tigers and leopards using spatially explicit capture‐recapture models (SECR) to further examine their coexistence mechanism in light of the recent recovery of tiger populations in PNP. In general, our findings indicate that both species co‐detected at 44 camera locations, demonstrating that they spatially share habitats inside the park. However, leopards avoid peak tiger activity periods, which is likely to reduce competitive interactions. The SECR model estimated a leopard density of 3.09 individuals per 100 km^2^ whereas tiger density was 1.25 individuals per 100 km^2^ within the study area. The model‐averaged occupancy probability of leopards in PNP was 0.45 (CI: 0.30, 0.64). The normalized difference vegetation index (NDVI) had a strong correlation with leopard occupancy, while the tiger relative abundance index (RAI) had minimal impact, reflecting the importance of high‐quality habitats in protected areas for conserving both species. Conservation initiatives targeting to strengthen the tiger recovery plans should incorporate thorough studies of interspecific interactions between sympatric large carnivores like tigers, leopards, and their prey base on a fine‐grain scale to ensure effective management strategies.

## 1 Introduction

Apex predators play a critical role in maintaining ecosystem stability by influencing habitat use, dietary overlap, and mechanisms of coexistence (Li et al. 2023; Linnell and Strand 2000; Ripple et al. 2014). Understanding these mechanisms is essential for the long‐term conservation of large carnivores in the face of global population declines and increasing human‐large carnivore conflict (Datta et al. 2023; Li et al. 2023; Rodrigues and Roth 2023; Srivathsa et al. 2023; Strampelli et al. 2023). Given that many large carnivore populations are confined to protected areas (PAs) that are relatively small in size, there is a growing need for research identifying the underlying factors facilitating their co‐occurrence within PAs and the surrounding multi‐use landscapes (Chatterjee et al. 2023; Lamichhane et al. 2019). In Nepal, only a few PAs support large carnivore populations that are demographically viable (Dahal 2023; Shah et al. 2024). However, limited human activities and high prey densities facilitate the occurrence of both large‐ and medium‐sized predators in a few PAs of Nepal's Terai region (Bisht et al. 2019; Lovari et al. 2015; Wegge et al. 2009). Thus, to retain large carnivore populations, it is essential to maintain protected area networks that are interconnected by ecological corridors (Alagador et al. 2012; Hilty et al. 2020; Vlková et al. 2024).

Tigers ( *Panthera tigris* ) and leopards ( *Panthera pardus* ) are the two big sympatric felids in Asia having a preference for similar habitat and diet (Goodrich et al. 2022; Jacobson et al. 2016; Simcharoen et al. 2014; Stein et al. 2023), which may lead to competition for limited resources (Karanth and Sunquist 2000; Kumar et al. 2020; Lovari et al. 2015; Odden et al. 2010; Selvan et al. 2013; Wegge et al. 2009). Both species are obligate carnivores and solitary hunters with similar morphology, differing mainly in body size; leopards are about a quarter the weight of tigers (Seidensticker 1976; Stein et al. 2023). Tigers are a socially dominant predator that influences temporal activity patterns (Chatterjee et al. 2023; Fedriani et al. 1999; Karanth and Sunquist 2000; Lamichhane et al. 2019; Pokheral and Wegge 2019), habitat use, and population size of other co‐existing carnivores including leopards (Bíl et al. 2021; Harihar et al. 2011; Karanth and Sunquist 2000; Odden et al. 2010; Seidensticker 1976; Sunquist and Sunquist 1989; Sunquist 1981).

Studies suggest that behavioral factors such as prey selection govern the coexistence mechanism between tigers and other sympatric carnivores (Johnsingh 1992; Karanth and Sunquist 2000, 1995; Wang and Macdonald 2009; Wegge et al. 2009). Some studies also report that large carnivores are responsible for the reduction of smaller predators through interspecific interactions (de Oliveira and Pereira 2014; Prugh and Sivy 2020; Ritchie and Johnson 2009). However, interactions can vary depending on predator guild composition, habitat composition, and resource availability (Ritchie and Johnson 2009). The scale of study also influences our understanding of species interactions; the broad‐scale research may overlook interactions occurring at finer spatial scales. Therefore, it is important to study interspecific interactions at smaller scales to discern fine‐scale niche partitioning and behavioral adaptations among competing predators in multi‐predator systems, which may not be apparent at broader scales (Durant 1998; Durant et al. 2010; Farris et al. 2020; Swanson et al. 2016).

Tiger and leopard interactions have been extensively studied in well‐known reserves like Nagarahole National Park, Rajaji National Park and Chitwan National Park (CNP) in South Asia (Harihar et al. 2011; Lamichhane et al. 2019; Li and Wang 2022), but their interactions in other areas of overlapping range particularly in those areas with recent tiger population colonization remains uncertain. Spatial segregation between tigers and leopards has been documented in some areas. However, in areas with high tiger density, tigers may outcompete leopards, forcing them towards peripheries of protected areas due to the ecological dominance of tigers over leopards (Harihar et al. 2011; Lovari et al. 2015; Odden et al. 2010; Pokheral and Wegge 2019; Steinmetz et al. 2013). This shift in leopard distributions towards PA peripheries increases the risk of livestock predation and human‐wildlife conflict (Bhattarai and Kindlmann 2012; Carter et al. 2015; Joshi et al. 2025). However, coexistence is possible in habitats with abundant prey and low population densities of tigers and leopards, despite spatial and temporal overlap (Lamichhane et al. 2019; Mondal et al. 2012). In such landscapes intra‐guild competition can alter feeding behavior, causing subordinate members to change their activity patterns to avoid dominant predators (Katuwal et al. 2024; Lamichhane et al. 2019; Lovari et al. 2015; Mondal et al. 2012; Pokheral and Wegge 2019). Therefore, numerous extrinsic landscape factors (such as vegetation and elevation) and prey resources (Karanth et al. 2017; Kumar et al. 2020), as well as intrinsic factors pertaining to the biology and foraging ecology of each species, are likely to have an impact on the coexistence mechanisms (Li et al. 2023; Steinmetz et al. 2013). Although several studies indicate that coexistence patterns between tigers and leopards vary across areas (Karanth et al. 2017; Karanth and Sunquist 2000; Lovari et al. 2015; Odden et al. 2010; Selvan et al. 2013; Wegge et al. 2009), little is known about the underlying factors that affect these coexistence mechanisms on a finer scale (Li et al. 2023; Thapa et al. 2021), and especially within the critically important Chitwan‐Parsa‐Valmiki forest complex of the Terai Arc Landscape (TAL).

Protected areas are fundamental to safeguard natural ecosystems and play a vital role in curbing the global decline of biodiversity (Bruner et al. 2001; Rodrigues et al. 2004), including the conservation of large carnivores (Van der Weyde et al. 2018; Wolf and Ripple 2018). Parsa National Park (PNP), situated within the Chitwan–Parsa–Valmiki forest complex of Nepal's TAL, is one such protected area contributing to the conservation of tigers and leopards. Despite its protected status, PNP face ecological limitations, including scarce water sources and low prey density, which reduce its capacity to support viable populations of large carnivores and potentially function as a sink for individuals dispersing from nearby source habitats (Lamichhane et al. 2018). A recent studies have documented that tigers in PNP are primarily exhibited crepuscular and nocturnal behavior, with activity concentrated in grassland, riverine and mixed forest habitats (Maharjan et al. 2024). Similar to this, Katuwal et al. (2024) reported that tigers exhibited predominantly nocturnal behavior whereas leopards showed diurnal behavior in PNP and adjoining habitats of Parsa‐Koshi Complex. Concurrently, increased conservation efforts, including the relocation of human settlements from park's core area and initiatives to restore habitat quality, have contributed to a notable recovery of tiger population in PNP (DNPWC and DFSC 2022; Lamichhane et al. 2018). As tiger density increases, leopards may be displaced towards the park's periphery (Odden et al. 2010) or compelled to alter their temporal activity patterns to minimize direct encounters with tigers (Katuwal et al. 2024; Lamichhane et al. 2019; Mondal et al. 2012). However, the extent to which leopards have adjusted their spatial and temporal behavior in response to growing tiger populations within PNP remains largely unknown.

Using camera trap data from across PNP, we examine how tigers and leopards exhibit spatial and temporal coexistence, particularly in the context of recent recovery of the tiger population and ongoing conservation efforts implemented in PNP in recent years. We hypothesized that if interference competition exists between tigers and leopards in PNP, leopards, as subordinate predators, will exhibit both spatial and temporal avoidance of areas and time periods with peak tiger activity. We predicted that, given their status as the dominant predator, tigers would display minimal spatial and temporal avoidance of leopards, but that leopards, as subordinate predators, would exhibit reduced activity during peak tiger activity periods and avoid areas frequently utilized by tigers to minimize competitive interactions.

## 2 Materials and Methods

### 2.1 Study Area

**FIGURE 1:**
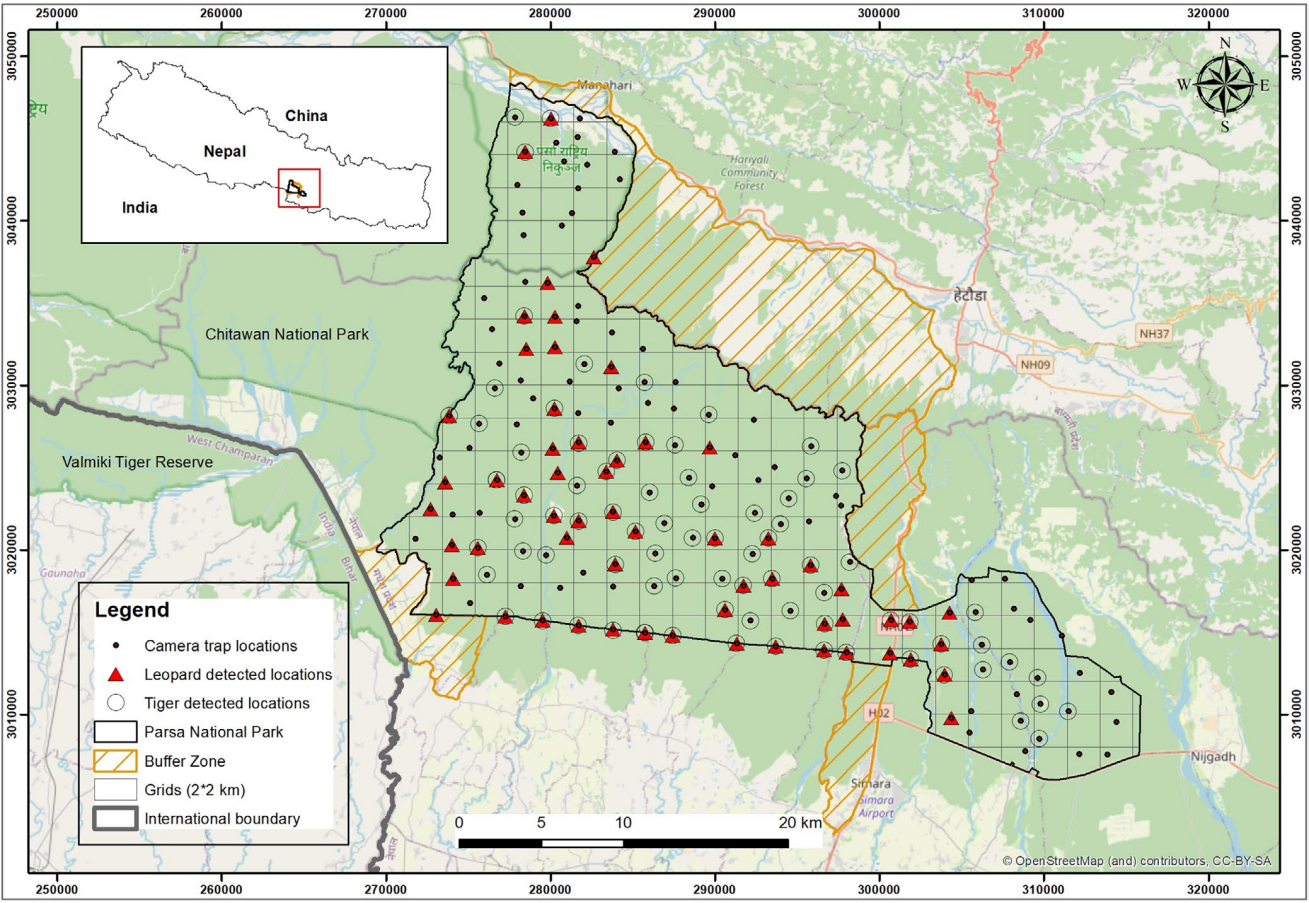
Study area map showing camera trap locations in 2 × 2 km grids within Parsa National Park. The black dots indicate camera trap deployment locations, while black circles indicate tiger detected locations and red triangles indicate leopard detected locations.

The study was carried out in Parsa National Park (PNP), situated in south‐central lowland of Nepal, spans from 27°15′ N to 27°33′ N and 84°41′ E to 84°58′ E (Figure 1). PNP is the easternmost protected area of the TAL, extended over 627.39 km^2^. It shares boundaries with Chitwan National Park (CNP) to the west and India's Valmiki Tiger Reserve (VTR) to the south‐west India. PNP predominantly consists of Churia hills, the outermost foothills of the Himalayas with elevations ranging from 750 to 950 m east to west, and the Bhabhar regions, rugged and highly porous alluvial substrate dominated by the Sal ( *Shorea robusta* ) forest. Its climate is monsoonal humid, with the majority of annual precipitation occurring between July and October, followed by an 8‐month dry season from November to June. The vegetation is categorized as subtropical dry‐deciduous forest, with colonizing kans ( *Saccharum spontaneum* ) and thatch grass ( *Imperata cylindrica* ) on dry riverbeds and floodplains evolving to climax Sal forests on the Bhabhar region and hillsides. Beside leopards and tigers, PNP harbors diverse mammalian fauna, including sympatric carnivores such as dholes ( *Cuon alpinus* ), striped hyenas ( *Hyaena hyaena* ), and golden jackals ( *Canis aureus* ). The park also supports a variety of wild prey species for both leopards and tigers, including gaur ( *Bos gaurus* ), nilgai (*Boselaphus tragocamelus*), sambar ( *Rusa unicolor* ), barking deer ( *Muntiacus muntjak* ), spotted deer ( *Axis axis* ), and wild boar ( *Sus scrofa* ) (DNPWC and DFSC 2022; Lamichhane et al. 2018; Thapa et al. 2014). The park holds relatively lower prey ungulate densities [75 (±SE 11.4) individuals per km^2^] compared to other protected areas in the region (DNPWC and DFSC 2022). Two settlements, such as Rambhori Bhata (74 ha, 96 households) and Ramauli Pratappur (116 ha, 377 households), both located in the core area of the park, were recently relocated outside the park from 2009 to 2013 (Lamichhane et al. 2018; Thapa et al. 2014). This event is anticipated to facilitate the recovery of the park's large carnivore populations, highlighting the importance of our study as a baseline for future research on interspecific interactions and coexistence mechanisms among sympatric carnivores.

### 2.2 Data Collection and Analysis

#### 2.2.1 Camera Trapping

The study area was systematically surveyed using paired motion sensor camera traps within 2 × 2 km grids throughout PNP (Figure 1). The placement of camera traps within each grid cell was carefully selected in accordance with comprehensive preliminary field surveys for both tigers and leopards, where species presence signs such as pugmarks, scats, and scrape marks were detected. Following these initial field surveys, two paired camera traps (Panthera V5 and V6 model with white flash) were systematically deployed at 157 locations, considering features like fire lines, trails, riverbanks, ridgelines, and forest roads (DNPWC 2017; Karanth and Nichols 2017) during the dry winter season (November 21, 2016 to February 12, 2017). To capture both flanks and the full body coverage of tigers and leopards, two camera traps were mounted on wooden posts or tree trunks at a height of 45 cm above ground level and spaced 3.5–6 m apart on either side of those strategic locations. These cameras were set up to shoot three pictures per trigger with no capture delay, and they were in operation for at least 3 weeks (ranging from 22 to 27 days) in each survey grid. Regular maintenance, such as camera checks, was performed on each camera trap to ensure functionality, and images and metadata were retrieved weekly. Due to limited availability of cameras, the survey area was systematically divided into three successive blocks, with each block surveyed one after another.

#### 2.2.2 Data Processing and Statistical Analysis

Photographs of tigers and leopards were organized from the raw data and segregated into separate folders based on camera trap locations. Individual tigers were visually identified through a thorough examination of all available images by field technicians and wildlife biologists from the National Trust for Nature Conservation‐Biodiversity Conservation Center (NTNC‐BCC). Individual tigers were identified based on their unique stripe patterns visible in “both flank” photographs and either right or left flank photographs at each survey site to ensure individual uniqueness. Only tigers (> 1 year old, those detected independently without their mother) were included in the analysis (Karanth and Nichols 1998). Similarly, leopards were identified using their distinctive pelage patterns, which are unique on each flank, facilitating individual identification (Miththapala et al. 1989). Accordingly, individual leopards were identified through visual comparison of pelage patterns from “both flanks” photographs and either right or left flanks based on their shape, size, and structure (Borah et al. 2014; Harihar et al. 2011; Thapa et al. 2014).

We used time‐stamped camera data to assess the activity patterns and activity overlap between leopards and tigers using the “overlap” package in program R applying kernel density functions to animal detections (Ridout and Linkie 2009; R Core Team 2024). A photo‐capture was considered as an independent event only if the same individual tiger or leopard was photo‐captured at the same camera location after an interval of 30 min (O'Brien et al. 2003). We estimated the coefficient of overlap (^Δ_1_) between leopards and tigers, where “0” indicates no overlap and “1” indicates complete overlap. Leopard and tiger detections at stations of co‐occurrence were aggregated to generate density curve for each species at stations of overlap. The coefficient of overlap was considered “low” if < 0.5, “moderate” if between 0.5 and 0.75, and “high” if > 0.75 (Monterroso et al. 2014). We calculated the 95% confidence interval for the coefficient of overlap by bootstrapping 10,000 samples from the species specific density curves (Bu et al. 2016).

To examine the potential effect of tigers and environmental covariates on detection (*p*) and occupancy (*Ψ*) of leopards, we conducted a single‐species single‐season occupancy analysis using 5‐day sampling occasions within the “unmarked” package in program R (Fiske and Chandler 2011; R Core Team 2024). Due to limited repeat of tiger detections across camera locations, we were not able to run two‐species occupancy models incorporating both leopard and tiger occupancy, as tiger occupancy models failed to converge. In our single‐species occupancy models, we included tiger capture history as a detection covariate (affecting day‐to‐day leopard detections) and tiger relative abundance index (RAI) as an occupancy covariate (affecting overall leopard occupancy). Tiger RAI was computed by dividing the total number of tiger detections at a station by the number of camera trap nights at that station, multiplied by 100. Similarly, we extracted distance to water (m), distance to human settlement (m), proportion of forest cover within 500 m radius of each camera trap locations using ESRI 2019 land use/land cover V2 at 10‐m resolution (Karra et al. 2021) and normalized difference vegetation index (NDVI) using Landsat Image 8 (https://earthexplorer.usgs.gov/) as a measure of vegetation productivity employing QGIS (QGIS Development Team 2023) and incorporated these as habitat covariates in the leopard occupancy modeling. We tested for correlations among all covariates and excluded distance to road due to its high correlation with distance to human settlement (*r* = |0.68|). The global occupancy model was assessed for over‐dispersion using the Mackenzie‐Bailey goodness‐of‐fit test (MacKenzie and Bailey 2004). To determine the best‐fitting leopard detection and occupancy model, we used the dredge function in the “MuMIn” package in program R to run all combinations of covariates and rank the resulting models based on Akaike Information Criterion adjusted for small sample sizes (AIC_c_) (Barton 2024; R Core Team 2024).

In addition, we estimated the density of leopards and tigers in PNP using spatially explicit capture‐recapture (SECR) methods using the “secr” package in program R (Efford 2024a; R Core Team 2024). We computed spatial detection histories using identified individual leopards and tigers compiled from camera trap photographs. To calculate *σ* (spatial scale of detection) for each species, we defined a habitat mask by delineating a buffer around each camera trap site using the Root Pooled Spatial Variance (RPSV) function in “secr” package. We then calculated 4*σ* to determine the final mask buffer width for each species as suggested by Efford (2024b). We generated separate habitat masks for leopards and tigers given their differing movements throughout the study period and fit SECR models without covariates for each species to determine densities.

## 3 Results

**FIGURE 2:**
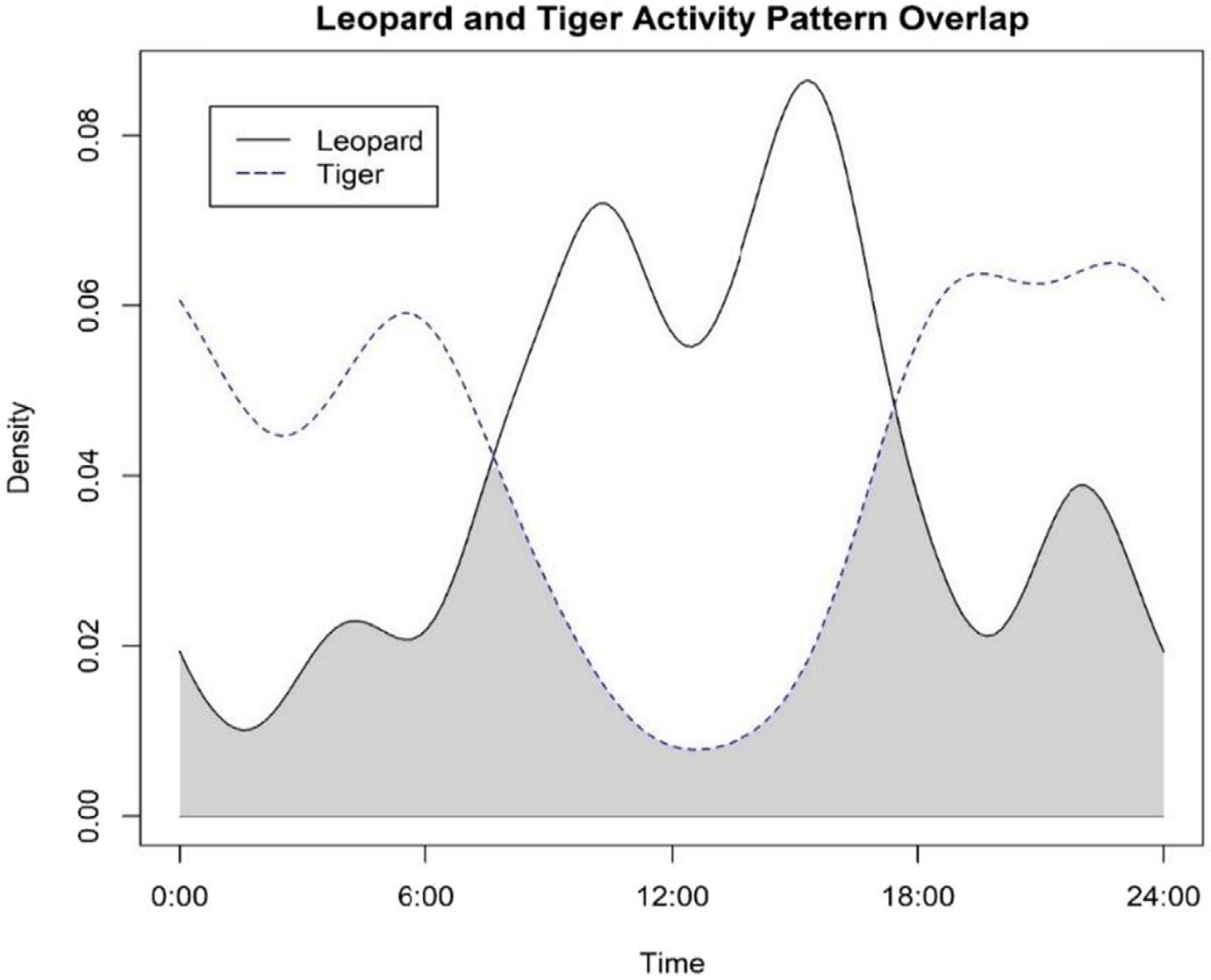
Temporal activity overlap of leopards with tigers at camera trap stations of co‐occurrence of both species in Parsa National Park, Nepal, November 2016–February 2017. Density represents the activity of species as measured by fitting the kernel density functions of animal observations.

We obtained 241 independent photographs of 17 individual tigers from 82 camera trap locations and 136 independent leopard photographs of 35 individual leopards from 70 camera trap locations across PNP over 3698 trap nights. Leopards and tigers were co‐detected at 44 out of 157 camera locations (Figure 1). At locations of co‐occurrence, leopards and tigers displayed moderate activity pattern overlap [Δ_1_ = 0.535, 95% CI (0.405, 0.664)] and appeared to display inverse activity patterns (Figure 2). Leopards exhibited more diurnal activity than tigers, with leopard activity increasing ~06:00 to peak at ~15:00 and then decreasing at dusk and night. In contrast, tigers were least active during the day and increased their activity at dusk (~18:00) to remain active throughout the night.

The global occupancy model's goodness‐of‐fit test revealed no evidence of lack of fit or overdispersion (c‐hat = 0.75). The mean detection of leopard was 0.25 [95% CI (0.19, 0.30)] and leopard occupancy across the study area was 0.45 [95% CI (0.30, 0.64)], respectively. The top leopard occupancy model included NDVI and tiger RAI. NDVI had a significant, positive effect on leopard occupancy [0.67, 95% CI (0.189, 1.15), *p* = 0.006]. Although tiger RAI was included in the best‐fit model, its effect on leopard occupancy was not significant (*p* = 0.13), and the estimates (95% confidence interval) overlapped with 0, indicating that the tiger RAI covariate was uninformative [0.37, 95% CI (−0.11, 0.85)]. Four additional models were within 2ΔAIC_c_ of the top model (Table 1). The second best‐fit model included only NDVI and had a similar model weight to the top model (top model weight = 0.12 vs. NDVI model weight = 0.10).

**TABLE 1:** Top occupancy (*Ψ)* models for leopards in Parsa National Park.

Model	*β* (settle)	*β* (water)	*β* (forest)	*β* (NDVI)	*β* (tiger)	AIC_c_	Δ AIC_c_	Weight
*p*(.) *Ψ* (NDVI + tiger)				0.67	0.37	623.57	0.00	0.12
*p*(.) *Ψ* (NDVI)				0.72		623.79	0.22	0.10
*p*(.) *Ψ* (settle + NDVI + tiger)	−0.31			0.58	0.38	624.60	1.03	0.07
*p*(.) *Ψ* (settle + NDVI)	−0.30			0.64		624.94	1.37	0.06
*p*(.) *Ψ* (water +NDVI + tiger)		0.12		0.63	0.39	625.51	1.94	0.04

*Note:* Parameter estimates for the occupancy covariates distance to human settlement (settle), distance to water (water), proportion of forest cover within 500 m radius from camera stations (forest), normalized difference vegetation index (NDVI), and relative abundance of tigers (tiger) are reported. AIC_c_ (Akaike Information Criterion corrected for small sample sizes), Δ AIC_c_, and weight of the top models are also reported.

**FIGURE 3:**
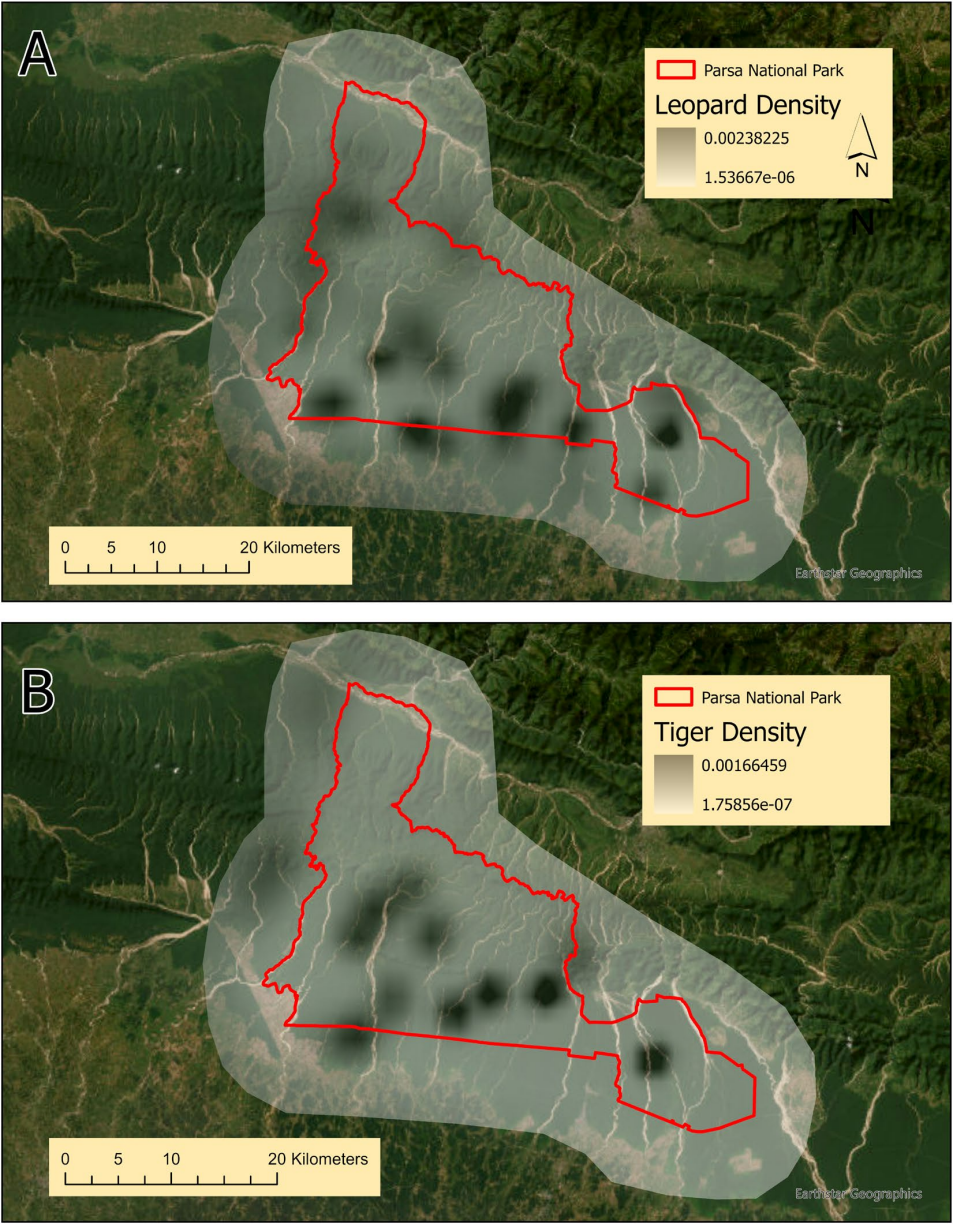
Map showing SECR‐based leopard (A) and tiger density (B) within Parsa National Park, Nepal, with pixel size ~1356 m, which comes from the average distance between cameras that is required to create the habitat mask and resulting density surface for tigers and leopards in SECR.

The habitat mask buffers generated surrounding the camera trap locations for leopards and tigers were ~9876.98 and ~11,386.56 m, respectively. The SECR model estimated a leopard density of 3.09 individuals per 100 km^2^, while tiger density was estimated to be 1.25 individuals per 100 km^2^ within the habitat mask buffer area in the study area (Figure 3).

## 4 Discussion

This study investigated the spatio‐temporal interactions of two sympatric large predators that play a crucial role in maintaining ecosystem stability in Asian forests (Li et al. 2023; Linnell and Strand 2000; Ripple et al. 2014). Using camera trap data from across PNP in Nepal, our findings indicate that both tigers and leopards co‐detected at 44 camera trap sites (Figure 1). However, they appear to reduce interference competition by adopting distinct activity patterns. This temporal segregation likely enables leopards to access shared resources during the periods of reduced tiger activity, thereby minimizing the risk of direct encounter with tigers (Chatterjee et al. 2023). This behavior is consistent with various studies showing leopards actively avoid the peak activity periods of tigers, particularly in prey limited habitats (Karanth et al. 2017; Karanth and Sunquist 2000; Lamichhane et al. 2019; Li et al. 2019; Mondal et al. 2012; Ramesh et al. 2012). Leopards' ability to adjust their activity patterns in response to tiger presence demonstrates their behavioral flexibility and the ecological pressures exerted by competition for shared resources. Understanding these interaction dynamics is essential for conservation efforts aiming to maintain viable populations of both species in overlapping habitats.

Studies from Nepal's protected areas, including CNP (Seidensticker 1976), Bardia National Park (Odden et al. 2010), and Shuklaphanta National Park (Lovari et al. 2015), which collectively elucidate the mechanisms of co‐occurrence at a fine‐grain scale, indicate that habitat partitioning is influenced by the behaviors of sympatric carnivores over time and space as well as their dietary preferences. Although diet and prey density were not incorporated into our study, we found that both tigers and leopards were co‐detected at many camera sites inside the park. Detecting co‐occurrence at trap sites (Figure 1) does not reflect spatial/habitat partitioning; however, temporal avoidance is visible from the results. We found moderate temporal overlap in leopard and tiger activity patterns, with leopards being more diurnal and tigers being more crepuscular in nature (Figure 2). Similar findings were reported from a sign‐based occupancy study conducted by Thapa et al. (2021) across the multi‐used Terai Arc landscape where leopard and tiger co‐occurrence were mediated by the availability of rich habitat and prey. A camera trap study conducted within the Parsa‐Koshi complex (including the PNP and the surrounding buffer zone) by Katuwal et al. (2024) also documented a positive association between leopard and tiger occupancy and moderate temporal overlap between leopard and tiger activity patterns. A similar camera trap‐based study by Maharjan et al. (2024) found that tiger occupancy was higher within certain habitat types within PNP, and they displayed crepuscular activity patterns. In contrast, a camera trap study conducted in CNP by Kafley et al. (2019) and Joshi et al. (2025) in Shuklaphanta National Park found spatial avoidance between these two predators at a fine scale. This suggests that prey availability, terrain conditions, and other habitat resources influence the co‐occurrence mechanisms. Therefore, this study is indicative of the fact that coexistence between tigers and leopards within a PA in South Asia, which is recovering from the negative effects of anthropogenic disturbance, was aided by the availability of habitat and most likely wild prey rather than the antagonistic interactions driven by intense interspecific competition. Given that our data were derived from a single‐season camera trapping, multi‐season camera trapping data and thorough studies on the diet and niche partitioning of these two sympatric predators would have been beneficial to better understand their interspecific interactions following the recent recovery of tigers and ongoing conservation efforts in PNP.

Occupancy model estimates showed that NDVI has a positive influence on leopard occupancy in PNP. This finding is consistent with the other studies conducted in Nepal's TAL region, which indicated that leopard occupancy declined in degraded habitats outside of PAs due to anthropogenic pressures such as deforestation and the presence of district roads (Lamichhane et al. 2021; Thapa et al. 2021). Furthermore, a study conducted in the Chure region of Nepal demonstrated a positive association between PAs and leopard occupancy. In contrast, no significant relationship was found between NDVI and leopard occupancy (Lamichhane et al. 2021). The coexistence of tigers and leopards in the PNP may be largely due to prey density and the recent efforts of the government and other conservation partners on habitat improvements and other conservation initiatives (Katuwal et al. 2024; Maharjan et al. 2024). Although the best‐fit model included the “tiger RAI” covariate, it was not statistically significant, indicating that tiger abundance does not strongly influence leopard occupancy in PNP. We acknowledge that limited repeated detections of tigers across camera trap sites prevented to run two‐species occupancy models. As a result, the tiger occupancy models failed to converge, making it unfeasible to incorporate two species occupancy model in the analysis.

This study revealed that the SECR model estimates of leopard density were 3.09 leopards per 100 km^2^ in PNP, which is similar to estimates reported by Thapa et al. (2014) in the Bhabar area of PNP (3.48 leopards per 100 km^2^). This finding is also comparable to the previous study in the Churia region of CNP (3.45 leopards per 100 km^2^) (Thapa and Kelly 2017). However, this density is significantly higher than that reported outside of PAs, specifically in the Kamdi biological corridor of western TAL (1.50 leopards per 100 km^2^) (Kandel et al. 2020). Additionally, when compared to other PAs with similar habitat conditions at a regional scale, our study is inline with the findings of Thapa et al. (2014) suggest other PAs may support much higher leopard densities than PNP. For instance, in Sariska Tiger Reserve, India, leopard density was reported to be 5.8 leopards per 100 km^2^ (Mondal et al. 2012), while in Mudumalai Tiger Reserve, India, it was notably higher at 13.17 leopards per 100 km^2^ (Kalle et al. 2011), and in Kumana National Park, Sri Lanka, it was significantly higher at 41 leopards per 100 km^2^, where tigers are absent (Rodrigo et al. 2025). This variability in leopard densities across South Asian PAs most likely reflects variance in prey availability, habitat conditions, and leopards' broad niche width in forested landscapes. Availability of sufficient prey biomass could be the major factor for supporting moderate leopard densities in PNP (Karanth et al. 2004; Katuwal et al. 2024), where ungulate prey densities appeared at 22 (SE 3.8) and 75 (SE 3.8) individuals per km^2^ in 2018 and 2022, respectively (DNPWC and DFSC 2018, 2022).

Similar to leopards, our estimated tiger density in PNP of 1.25 tigers per 100 km^2^ (2016–2017), which is consistent with the densities, 0.92 (SD ±0.15) and 1.74 (SD ±0.17) tigers per 100 km^2^ reported in PNP and adjoining forests in 2018 and 2022, respectively (DNPWC and DFSC 2018, 2022), suggesting the potential growth of tiger population since the relocation of human settlements from the park's interior. However, tiger density in PNP remains low compared to the adjacent CNP, which recorded densities of 3.28 (SD ±0.19) and 4.06 (SD ±0.22) tigers per 100 km^2^ in 2018 and 2022, respectively (DNPWC and DFSC 2018, 2022). The current densities of tiger prey (medium and large‐sized ungulates) in PNP are most likely insufficient to sustain a high‐density tiger population, assuming an annual kill rate of 50 ungulates per tiger with an annual biomass cropping rate of 10% (Sunquist 1981). This could be the potential reason the park harbors low tiger density when compared to CNP, which may allow leopards to completely take advantage of their preferred medium and small‐sized prey, perhaps explaining the higher density of leopards in PNP (Karanth et al. 2004).

## 5 Conclusions

This study investigated the spatial and temporal dynamics of coexistence between tigers and leopards in PNP, Nepal, a landscape undergoing ecological recovery of tiger populations following intensified conservation efforts. Drawing from extensive camera trap data, we assessed how subordinate leopards respond to potential interference competition from tigers within a shared habitat. Although the two species co‐occurred in the shared habitats, leopards exhibited clear signs of temporal segregation, reducing their activity during periods of peak tiger movement. This behavioral adjustment backed leopards to mitigate direct competition with tigers through temporal avoidance, a strategy that may facilitate coexistence under increasing tiger densities. Recognizing leopards rely on temporal avoidance to mitigate competition with tigers and other super predators, such as humans, management plans should incorporate species‐specific activity data to guide park operations, such as scheduling human movement, patrolling, and infrastructure development to reduce interference with wildlife behavior. Our findings also revealed a strong association between NDVI and leopard occupancy, highlighting the critical role of high‐quality habitats in supporting leopard persistence within multi‐predator systems. Given the strong link between habitat quality and leopard occupancy, conservation policies should prioritize the restoration and maintenance of high‐quality habitats within PAs to sustain viable populations of these sympatric large carnivores. Future research should aim to explore evidence‐based interactions among sympatric large carnivores and their prey guilds across seasons and at a fine spatial scale to better inform conservation planning in Nepal and other regions experiencing similar predator recoveries.

## Author Contributions

**Dol Raj Thanet:** conceptualization (lead), data curation (equal), formal analysis (equal), methodology (equal), writing – original draft (lead), writing – review and editing (lead). **Pramod Raj Regmi:** conceptualization (equal), data curation (lead), investigation (equal), methodology (equal), writing – original draft (equal), writing – review and editing (equal). **Babu Ram Lamichhane:** methodology (equal), validation (equal), writing – review and editing (equal). **Dipanjan Naha:** conceptualization (equal), formal analysis (lead), writing – review and editing (equal). **Caitlin Kupferman:** conceptualization (equal), formal analysis (lead), writing – review and editing (equal). **James C. Beasley:** writing – review and editing (equal). **Mandip Pangeni:** data curation (equal), writing – original draft (equal), writing – review and editing (supporting). **Anil Shrestha:** writing – review and editing (equal). **Saneer Lamichhane:** writing – review and editing (equal). **Haribhadra Acharya:** methodology (equal), project administration (equal), writing – review and editing (equal). **Bed Kumar Dhakal:** methodology (equal), project administration (equal), writing – review and editing (equal). **Bhagawan Raj Dahal:** methodology (equal), project administration (equal), writing – review and editing (equal). **Chiranjibi Prasad Pokheral:** methodology (equal), validation (equal), writing – review and editing (equal). **Naresh Subedi:** methodology (equal), project administration (equal), validation (equal), writing – review and editing (equal).

## Conflicts of Interest

The authors declare no conflicts of interest.

## Data Availability

Data associated with this manuscript can be accessed at the Zenodo data repository (https://doi.org/10.5281/zenodo.14850176).
